# A Robust Closed-Tube Method for Resolving *tp53*^M214K^ Genotypes

**DOI:** 10.1089/zeb.2023.0030

**Published:** 2024-06-17

**Authors:** Katherine M. Silvius, Genevieve C. Kendall

**Affiliations:** ^1^ Center for Childhood Cancer Research, The Abigail Wexner Research Institute, Nationwide Children's Hospital, Columbus, Ohio, USA.; ^2^ Department of Pediatrics, The Ohio State University College of Medicine, Columbus, Ohio, USA.

**Keywords:** genotyping, HRMA, *tp53*, *tp53*^M214K^ zebrafish mutant, wild-type spike-in

## Abstract

The *tp53*^M214K^ zebrafish mutant is a versatile platform with which to model a diverse spectrum of human diseases. However, currently available genotyping methods for this mutant require lengthy hands-on processes such as restriction digests and outsourced Sanger sequencing. To address this deficiency, we leveraged high-resolution melting analysis technology in conjunction with a parallel, in-tandem wild-type spike-in approach to develop a robust genotyping protocol capable of discriminating *tp53*^M214K^ zygosity. In this study, we describe our method in detail. We anticipate that our genotyping protocol will benefit researchers utilizing the *tp53*^M214K^ zebrafish mutant by offering reliable results with a shorter turnaround time, lower personnel involvement, and higher throughput than traditional methods, thereby decreasing the burden of genotyping and maximizing research efficiency.

Animal models with *TP53* mutations are critical for the development of *in vivo* genetic systems that recapitulate the innate complexity of the human disease spectrum. *TP53* mutations are often required for animal models to form tumors, as demonstrated with the BRAF^V600E^-driven melanoma and PAX3-FOXO1 fusion-driven rhabdomyosarcoma zebrafish models.^1,2^ To these ends, the *tp53*^M214K^ zebrafish mutant developed by Berghmans et al.^3^ (also referred to as *tp53*^zdf1^) is widely used when building vertebrate disease models that require a *TP53*-mutated background.

This mutant is commercially available (ZIRC and ZL1057) and harbors a missense mutation in the DNA-binding domain of *tp53*, which mimics the most common type of *TP53* mutation found in humans.^3,4^ Specifically, the mutant allele contains a T > A point mutation that results in a change from methionine to lysine and consequential dysregulation of *tp53* function.

Since zebrafish are not in-bred, efficient genotyping is required to support research efforts. Current widely used approaches for genotyping the *tp53*^M214K^ mutant involve multiple hands-on steps, such as PCR purification and gel resolution. These lengthy processes are rate-limiting to genotyping scale and “hands on” time for personnel (Fig. 1A, B).^3,5,6^ In this study, we leverage high-resolution melt analysis (HRMA) technology to decrease the active time required for genotyping *tp53*^M214K^ mutants (Fig. 1C).

**FIG. 1.**
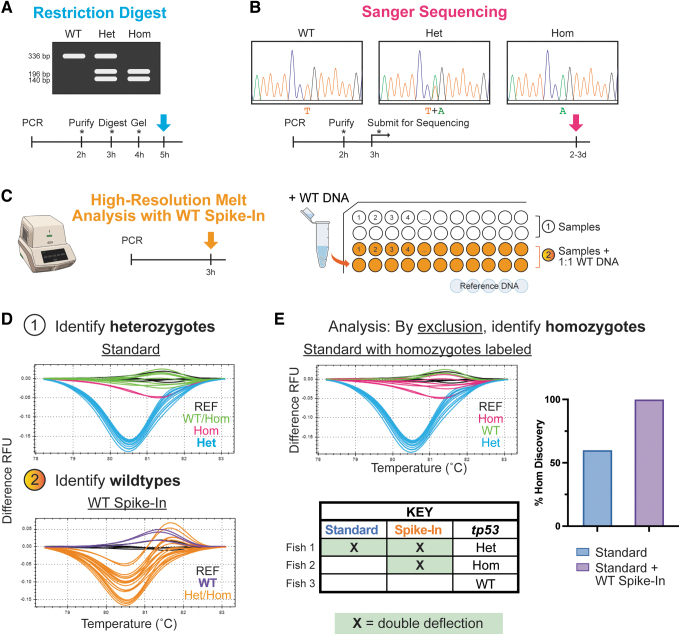
An improved workflow for *tp53*^M214K^ genotyping. Conventional genotyping protocols for the *tp53*^M214K^ mutant, including **(A)** restriction digest and **(B)** Sanger sequencing-based approaches are time-consuming and/or require multiple hands-on steps (indicated by *asterisk*), whereas our protocol uses **(C)** HRMA and requires a single PCR program on a standard qPCR machine (*left*); samples are run in parallel with and without WT DNA spiked in at a 1:1 ratio alongside a set of WT reference DNA for comparison (*right*). **(D)** The analysis strategy utilizes the characteristic deflection pattern of *tp53* heterozygotes to first identify heterozygotes in the standard run with 1 μL sample DNA; putative homozygotes that can be called with the standard HRMA are indicated in *pink* (*1*). Since WT DNA added to homozygotes yields a similar pattern to heterozygotes, samples that do not demonstrate that deflection pattern with the spike-in can be identified as WTs (*2*). **(E)** True homozygotes can then be identified by comparative exclusion as illustrated in the *table*. The *graph* shows all homozygotes (also verified by sequencing) in *pink* overlaid on the standard curve. When only the standard samples are evaluated, only ∼60% of homozygotes are called. However, when the parallel spike-in is analyzed, all homozygotes are correctly identified. Het, heterozygote; Hom, homozygote; HRMA, high-resolution melt analysis; qPCR, quantitative polymerase chain reaction; REF, reference DNA; RFU, relative fluorescence units; WT, wild-type.

HRMA is a technique that incorporates a gradual systematic heating step after amplification. Zygosity is then discriminated based on differences in DNA melting temperature of the mutant allele compared with a wild-type reference. HRMA requires access to a specialized PCR machine and associated software (e.g., No. 1845015; Bio-Rad). If possible to access these items, HRMA can greatly reduce the active steps required for genotyping, as reactions are performed in a closed tube, require minimal setup, and involve simple analysis.

Importantly, HRMA is sensitive enough to detect single-nucleotide polymorphisms (SNPs) and has been successfully utilized in zebrafish.^7–9^ Bertho et al.^7^ has previously used HRMA to genotype the *tp53*^M214K^ mutant. The authors developed a two-pronged approach whereby a secondary HRMA reaction with wild-type DNA spiked in identifies homozygote mutants. This alludes to the complexity of the allele; namely, a T > A point mutation that sits in a guanine and cytosine (GC)-rich region.

In this study, we present a detailed protocol, including the nuances of the assay (DNA isolation technique, DNA input, primer design, and rates of successful calls for a primary vs. spike-in assay) and specific guidelines for calling genotypes. As the *tp53*^M214K^ line is frequently used in the zebrafish community and considering the sizeable burden of line maintenance on personnel time, an optimized detailed HRMA genotyping protocol would offer an attractive alternative to researchers using this mutant.

To begin, we evaluated the viability of a single-step HRMA assay: we designed^9^ and tested a series of primer sets flanking the *tp53*^M214K^ mutation site with amplicon sizes ranging from 63 to 125 bp. Amplicons of <100 bp are often used for SNP scanning as small changes in the mutant allele are amplified when the total region is also small.^10^ However, we were not able to consistently differentiate *tp53*^M214K^ genotypes.

Heterozygotes were always clearly defined by a characteristic double deflection pattern, but repeated tests demonstrated that a small but persistent percentage of homozygotes often clustered with wild types (Fig. 1D). This is likely due in part to the fact that T > A transversion is associated with the smallest energy change of all possible point mutations, which inherently makes *tp53*^M214K^ zygosity more difficult to discriminate.^10^ In addition, as the primers move closer to the mutation site, the GC percentage continually increases (>60%), which precludes the feasibility of continuing to decrease the amplicon size.

To address this challenge, we took the four most promising primer sets (110, 96, 83, and 70 bp) and titrated the DNA input by serial dilution from 300 to 12.5 ng as measured by Nanodrop. Although this showed that the assay, particularly with the 96-bp primer, is able to detect all heterozygotes and most homozygotes in a working range of 10–200 ng DNA input, we were still unable to achieve the consistency necessary for a robust stereotyped genotyping protocol (Supplementary Fig. S1).

However, since our assay showed promise at a wide range of DNA concentrations, we decided to eschew normalization in further tests, as using a standard DNA volume rather than amount greatly reduces setup time. Although the concentration of tail-clip-derived DNA sample varies, we found that our DNA typically ranges from 100 to 400 ng/μL and is sufficient for use in most HRMA applications.^9,11^ Overall, this approach increases the throughput of the assay.

With a single-step HRMA effectively ruled out, we decided to test and characterize our 96-bp HRMA assay with a complementary wild-type spike-in approach similar to what was used previously.^7^ In this strategy, each sample is run twice on the sample plate, once with and once without wild-type DNA spiked in, to allow for genotype resolution by comparative exclusion.

For example, adding wild-type DNA will drop a homozygote's deflection to that of an easily identifiable heterozygote without affecting wild-type samples; true heterozygotes are identified from the run without the spike-in and are not affected by the spike-in, and then homozygotes can be discriminated by identifying samples that exhibit a double deflection pattern only after the addition of the spike-in DNA (Fig. 1D, E). We confirmed this by running samples with a total DNA volume of 1 μL, both with and without wild-type DNA spiked in using a 1:1 ratio. In masked tests without the spike-in, only ∼60% of homozygotes could be accurately called, but when the parallel spike-in was added, all genotypes were called correctly (Supplementary Fig. S2).

Altogether, we present a detailed HRMA-based genotyping protocol, which leverages a complementary in-tandem wild-type spike-in approach to accurately resolve *tp53*^M214K^ zygosity. We include this protocol in the Supplementary Data as a faster less-involved alternative to conventional digest and sequencing-based approaches. We also expect that a similar strategy could be employed to rapidly and efficiently genotype other challenging alleles.
