# Supplementary material for: A Robust Closed-Tube Method for Resolving tp53M214K Genotypes
Source: Zebrafish. 2024 Jun 17;21(3):250–4. doi: 10.1089/zeb.2023.0030 (PMC11296207; doi:10.1089/zeb.2023.0030)
Supplement: Supplementary Data [file zeb.2023.0030_suppl_data.docx]

**Supplemental Material**

**Genomic DNA Isolation**

We use a Proteinase K digestion to generate genomic DNA. The lysis step can be performed for as little as 1.5 hours to overnight to optimize the timing of experiments. Typical DNA concentrations by Nanodrop are around 100-400 ng/µL. The DNA quality is sufficient for HRMA applications, and a standard volume of 1 µL is used for this genotyping protocol (Supplemental Fig. 1). It is likely that other DNA isolation methods would also work with our high-resolution melt analysis (HRMA) protocol, but these would have to be validated by the user.

Proteinase K Genomic DNA Isolation Protocol

1. Anesthetize juvenile or adult zebrafish in 0.2 mg/mL Tricaine diluted in fish water and use a sterile razor to cut the tail fin just before the cleft where the tail bifurcates into the two lobes.
2. Transfer the tail-clips to a PCR strip tube or a 96-well plate.
3. Add 100 µL of Lysis Buffer (10mM Tris-HCl pH 8.3, 50mM KCl, 0.3% Triton X-100, 0.3% NP40) to each tail-clip.
   1. For 50 mL 1X Lysis Buffer: Add 500 µL 1M Tris-HCl pH 8.3, 2.5 mL 1M KCl, 150 µL Triton X-100, and 150 µL NP40 to 47 mL RO water. Shake and/or gently heat in a water bath to dissolve detergents. Store at 4˚C.
4. Transfer samples to a thermocycler and run program with a 95˚C lid: 98˚C x 10 min, 12˚C x 10 min, END.
5. Add 10 µL Proteinase K, 10 mg/mL, to each fin, flick or vortex to mix, then spin down.
6. Return samples to the thermocycler and run program with a 95˚C lid: 55˚C x 1.5-16 hours, 98˚C x 10 min, and HOLD at 12˚C.
7. Store DNA at 4˚C overnight or proceed directly to the HRMA protocol.

**Wildtype References**

HRMA requires samples to be compared to a set of known wildtype references in order to set the baseline melting profile for mutant allele detection. In these studies, AB fish were used as the reference since AB is the wildtype genetic background of the *tp53*^M214K^ line. Reference DNA is prepared with the same Proteinase K protocol described above and is also used as the DNA for the spike-in, where wildtype DNA and test DNA are added in equal measure. For the HRMA reference, we suggest that at least 5 known wildtype samples are run per plate so that up to 2 samples with an unexpected or abnormal curve in the reference cohort can be excluded without adversely affecting downstream analysis. Wildtype reference DNA can be collected in bulk using the standard practices described above and kept at -20˚C for longer-term storage.

**Instrumentation and Software Requirements**

- Real-time PCR machine: The HRMA software is compatible with all Bio-Rad CFX real-time PCR systems. Here, we used a CFX384. Each machine that will be used for HRMA must first be calibrated to ensure that the dye in the Precision Melt Supermix (Bio-Rad, #1725112) is correctly detected. This is done with the Melt Calibration Kit (Bio-Rad, #1845020) and only needs to be performed once per machine.
- CFX Maestro Software for Windows PC (Bio-Rad, #12013758): This program is only used to convert the run data from the PCR machine to a file type that can be processed using the HRMA software.
- Precision Melt Analysis Software (Bio-Rad, #1845015), 2 user licenses: The HRMA software is only compatible with Windows operating systems. There is also an available option that packages one calibration kit with the purchase of the software (Bio-Rad, #1845025).

**Note on Genotyping Call Efficiency**

When only the standard run with 1 µL DNA is evaluated, all heterozygotes are called with confidence. If only heterozygote mutant scanning is desired, running the standard set alone is sufficient. Homozygotes are more difficult to discriminate from wildtype from a single run without a spike-in control. Neither normalizing the input DNA amount (Supplemental Fig. 1) nor changing the size of the amplicon (data not shown) is able to mitigate this problem. Evaluating the standard set alone resolves only about 60% of homozygotes, on average. Homozygotes typically display a single deflection curve that falls below -0.05, but a subset persistently clusters with wildtypes (Supplemental Fig. 2A). The solution to this is using the standard and complementary wildtype spike-in protocol. When this strategy is implemented in parallel, 100% of homozygotes can be accurately resolved (Supplemental Fig. 2A-B).


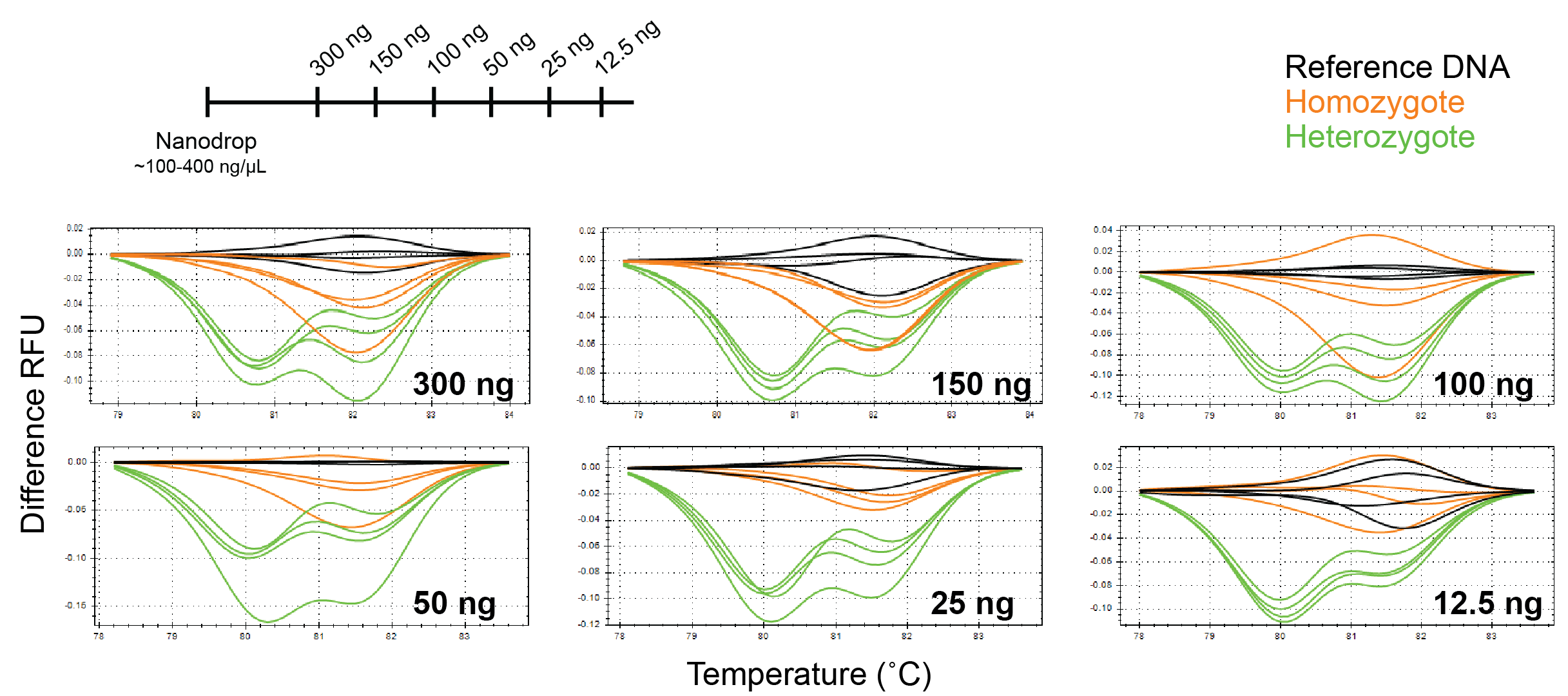


**Supplemental Figure 1. DNA concentration titration does not improve resolution of *tp53* zygosity.** Relative DNA concentrations of Proteinase K lysed DNA was measured by Nanodrop and serially diluted from 300 ng to 12.5 ng. Four known samples (unmasked) each of *tp53* wildtype (AB), *tp53*^M214K^ homozygotes, and *tp53* ^M214K^ heterozygotes were run (unmasked) at the following DNA inputs: 300 ng, 150 ng, 100 ng, 50 ng, 25 ng, and 12.5 ng. Each experiment was repeated 2-3 times. One representative melt curve at each DNA input with the 96-bp primer set is shown. While *tp53* heterozygotes can be called at every concentration with high confidence, *tp53* homozygotes and wildtypes often overlap which cannot be mitigated by simply normalizing the DNA concentration.


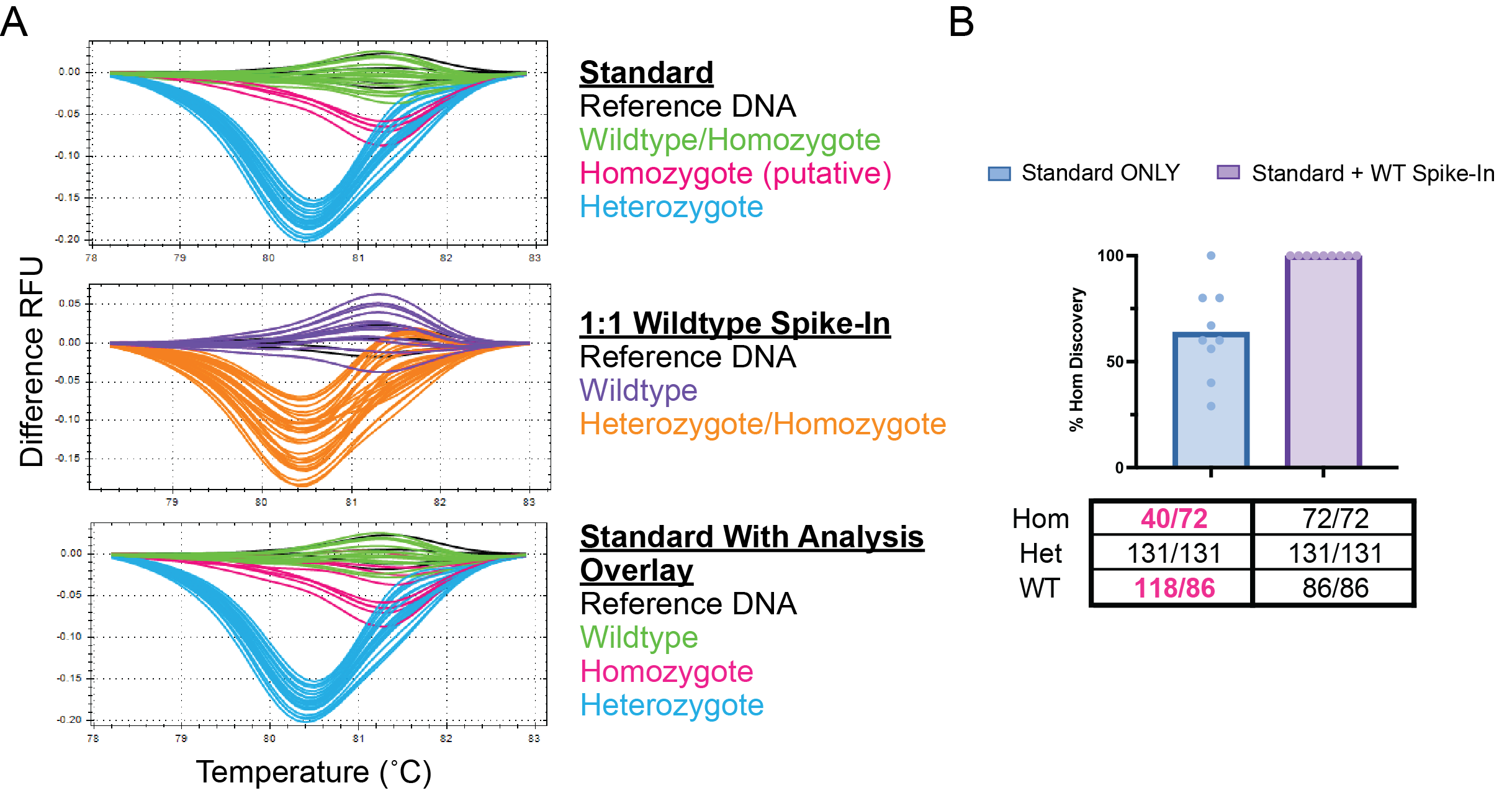


**Supplemental Figure 2. Complementary wildtype spike-in approach accurately resolves *tp53* genotypes.** **(A)** The melting curves for one representative experiment are shown. In this example, n=46 fish were genotyped in a masked manner using our HRMA protocol. The top two panels display the individual analyses for the standard (1 µL sample DNA) and wildtype spike-in (0.5 µL sample: 0.5 µL wildtype DNA), and the bottom panel overlays all homozygotes onto the standard melt curve. **(B)** These data are also presented in Figure 1E, with more specifics indicated in this panel. Each point represents one independent experiment with sample number ranging from 15 to 98 individual fish. The total numbers of correctly identified fish are reported in the table as a sum across the experiments represented in the graph. When analyzing only the standard run, the number of homozygotes is often underestimated (average: 60% of total) whereas the wildtype population is overestimated, denoted in pink. However, when the corresponding spike-in samples are evaluated in tandem, all calls are correct. Abbreviations: WT, wildtype; Het, heterozygote; Hom, homozygote.

**Detailed *tp53*^M214K^ HRMA Genotyping Protocol with Complementary Wildtype Spike-In**

Materials

- Precision Melt Supermix (Bio-Rad, #1725112)
- tp53 HRMA FWD Primer: GGACAACTGTGCTACTAAACTACATG
- tp53 HRMA REV Primer: CCTGAGTCTCCAGAGTGATGATT
- Proteinase K lysed genomic DNA from test samples and known wildtypes (latter used as reference DNA and for the wildtype spike-in)
- 384-well plate (Bio-Rad, #HSP3805)
- Microseal ‘B’ PCR Plate Sealing Film (Bio-Rad, #MSB1001)

Protocol

1. Prepare a master mix scaled appropriately to account for the total number of samples:

**total** = (# unknown) x 2 + (4-5 wildtype references) + 1 negative control + extras

| **COMPONENT** | **1X** |
| --- | --- |
| Precision Melt Supermix | 9 µL |
| tp53 HRMA FWD, 10µM | 0.5 µL |
| tp53 HRMA REV, 10µM | 0.5 µL |
| Nuclease-free water | 9 µL |

1. Add 19 µL master mix per well of a 384-well plate (note: 96-well plate would work too).
2. Briefly spin down all DNA samples in a mini-centrifuge.
3. For the wildtype references and the standard set of samples without the spike-in, add 1 µL DNA. For the corresponding set with the complementary spike-in, first add 0.5 µL sample DNA, then go back and add 0.5 µL wildtype DNA to each well. *Make sure to avoid any debris at the bottom of the genomic DNA preps.*
   1. Any of the wildtype reference samples can be used as the DNA for the spike-in. However, choose the same fish for the spike-in for the entire plate.
   2. Note: If wildtype spike-in DNA is pipetted incorrectly, homozygotes may be missed.
4. Cover the plate with B adhesive film and roll to completely seal. *Avoid touching the top.*
5. Briefly vortex and microfuge in a plate spinner.
6. Run the following HRMA program on a real-time PCR machine with 95˚C lid and 20 µL reaction volume: 95˚C x 3 min, (95˚C x 15 sec, 60˚C x 20 sec, Plate Read, 70˚C x 20 sec) x 44, 65˚C x 30 sec, melt curve 65˚C-95˚C with 0.2˚C/step for 5 sec plus Plate Read, 95˚C x 15 sec.


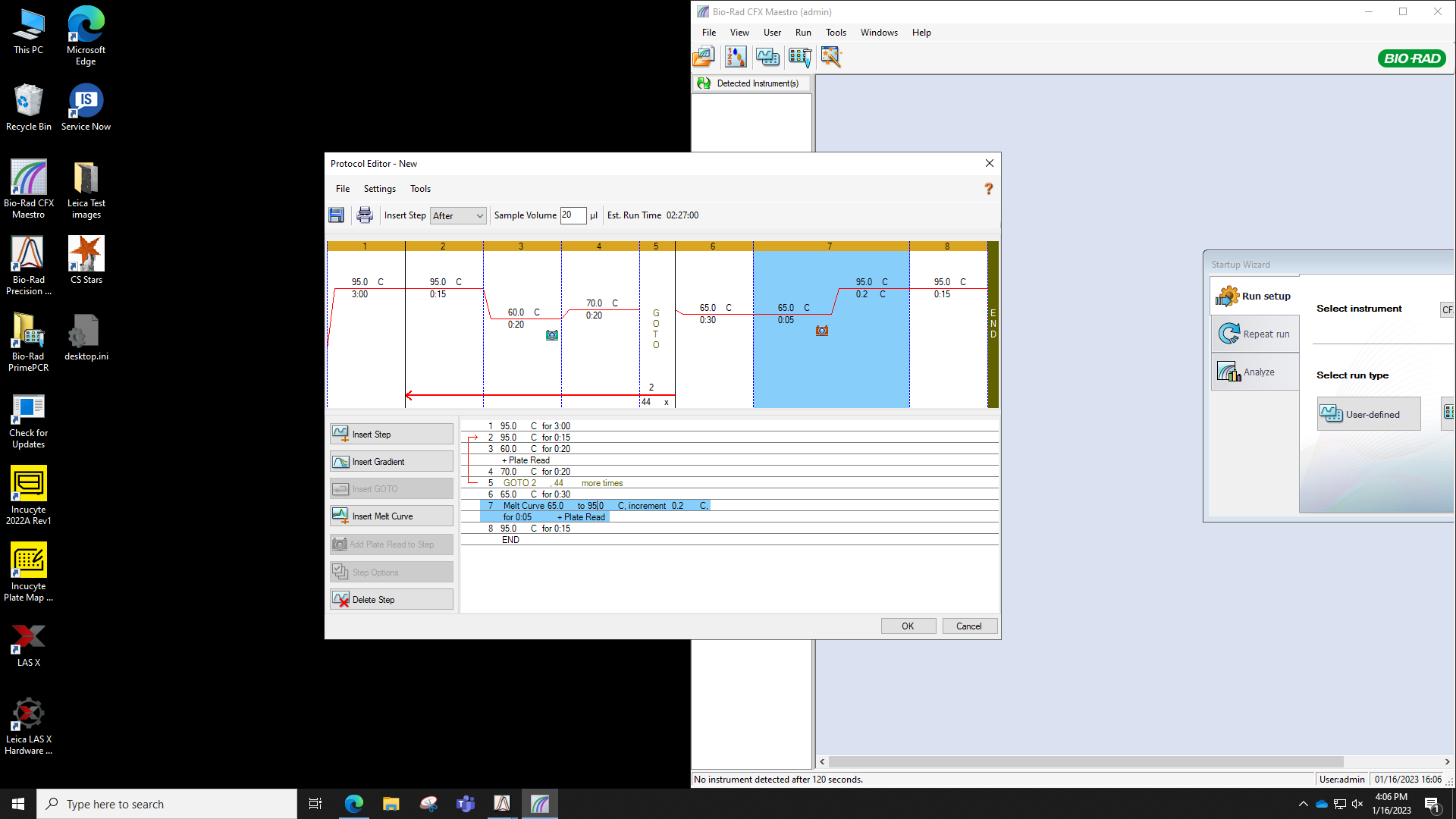


**Analysis**

1. Open the .ZPCR file of the run from the real-time PCR machine using CFX Maestro. This will convert the file to a .PCRD, which is the file type accepted by the HRMA software. The Cq value of the negative control can also be checked with Maestro. It should be “N/A” or greater than 38 to signify that there is no contamination.
2. Launch the Precision Melt Analysis software and open the .PCRD file.
   1. File → Open → New → Melt File (.PCRD file)
3. Click on the “View/Edit Plate” button in the toolbar to launch the plate editor view in a new window. This view will be used to exclude all samples except those that are actively being analyzed. This will ensure that the software is only considering the appropriate values that are relevant to the current step in the analysis process, which will prevent other samples from inappropriately influencing the deflection curves.
4. Select all wells **except** the wildtype references and the set of unknown samples without the spike-in. To do this, click and drag while holding the Control key to select the appropriate wells, scroll down on the right panel, and check the “Exclude Wells in Analysis” box (indicated with yellow arrow). Save the changes and exit the plate editor.


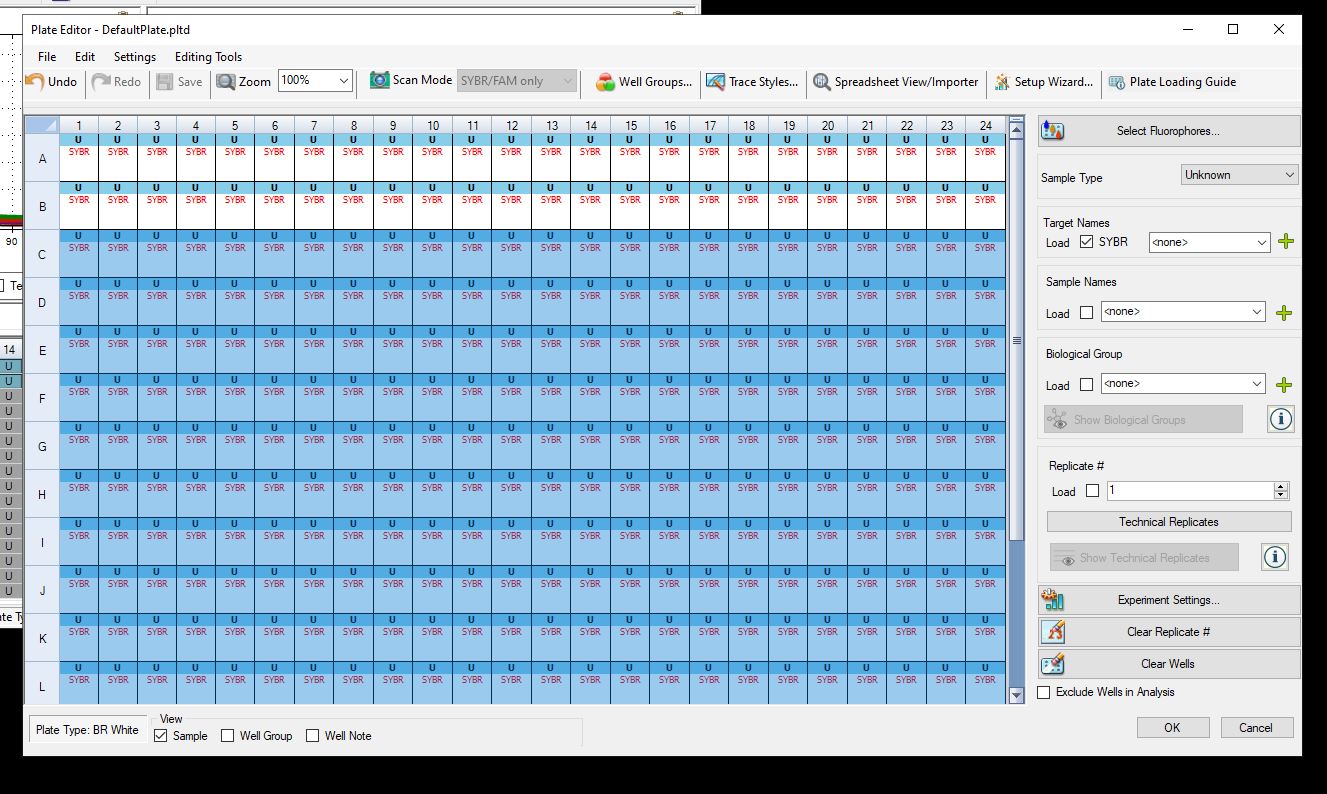


1. The program’s algorithm automatically assigns a reference population. To set the correct reference, gray out all the samples except for the wildtype references in the plate layout view on the bottom left by clicking and dragging to temporarily hide other samples.


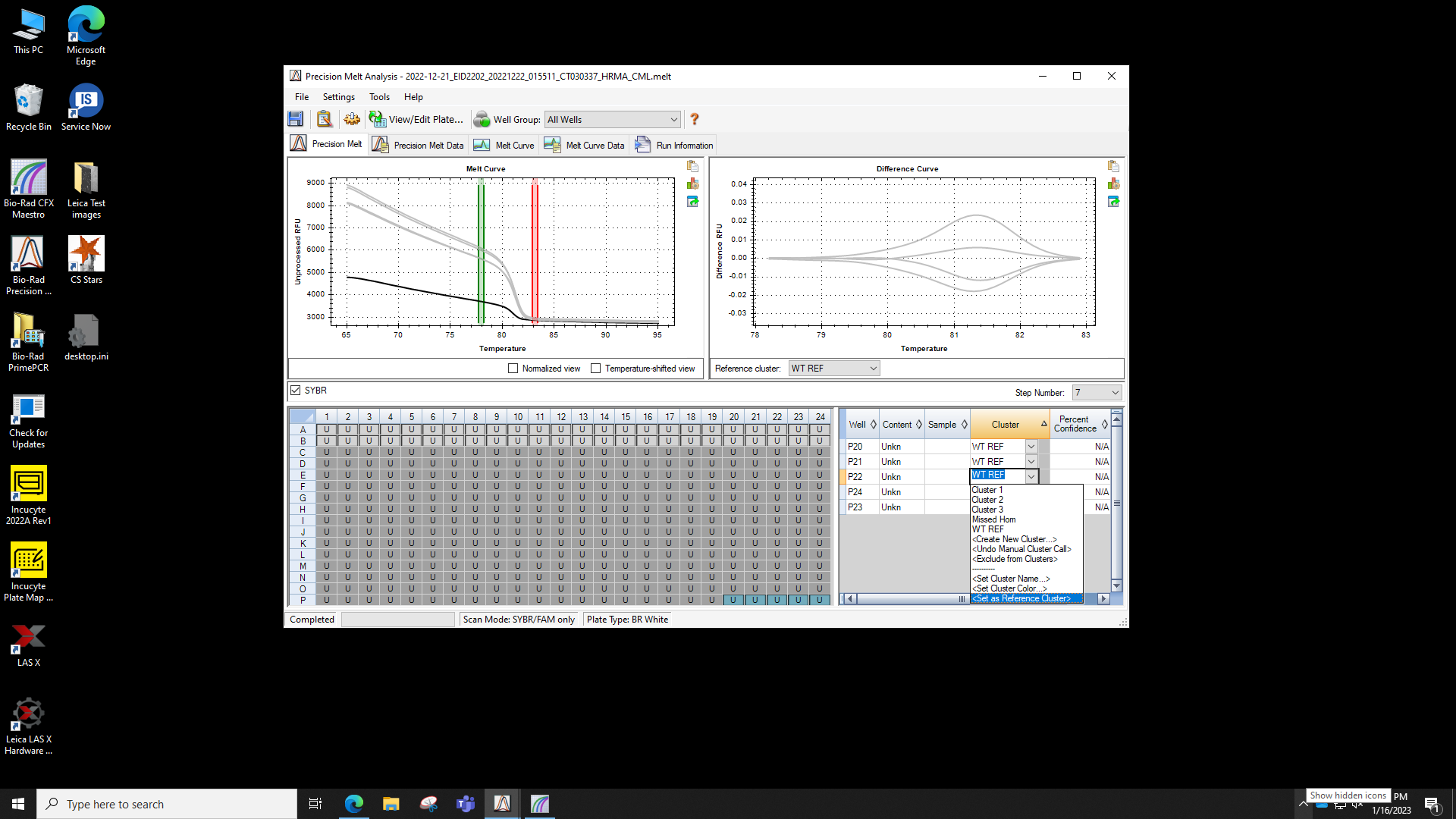


1. Click the dropdown next to the cluster name of one wildtype sample. Select “Create New Cluster” and assign a new name, e.g., *WT REF.* Then, click “Set as Reference Cluster.”
2. Using the same dropdown menu associated with the other wildtype samples and assign each to the same cluster as in Step 7.
   1. Exclude any obvious outliers. Including 5 wildtype reference samples on the plate allows for the exclusion of 1-2 outliers without impacting analysis.
3. Unhide the standard samples from Step 5 (click and drag). Now, the software’s algorithm will automatically assign clusters based upon commonality of melting point deviation from the selected wildtype references.

**Standard (1 µL sample DNA)**


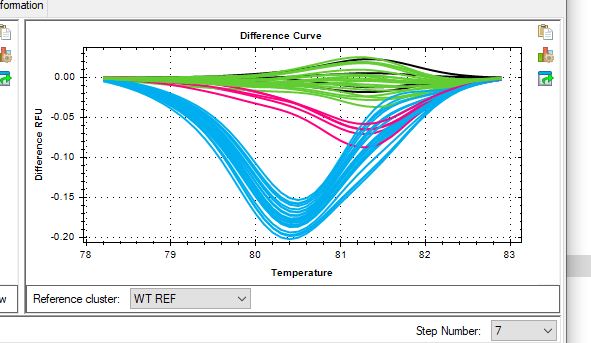


REF DNA

WT/Hom

Het

Hom (putative)

1. All samples with two deflections in the curve are **heterozygotes.** The software will not always group these samples together. Manually assign to the same cluster as needed. The rest of the samples are either wildtypes or homozygotes, which are not reliably distinguishable from one another with only the standard run.
   1. Note: A subset of homozygotes deflects lower than -0.05 (indicated in pink above; also see Supplemental Fig. 2). These may be called as putative homozygotes if desired.
2. (Optional) Create new separate clusters for heterozygotes, wildtypes/homozygotes, and putative homozygotes.
3. To analyze the deflection patterns of the *standard samples*, either:
   1. Hover over the samples in the plate set-up panel to highlight the corresponding deflection curve in the generated graph, OR
   2. Scroll through the samples on the bottom right panel.
4. Using a table similar to the example below, mark samples with a double deflection curve in the standard run, i.e., with an “X” or by coloring in the box. *Optionally, you may choose to call samples with a deflection amplitude lower than -0.05 as putative homozygotes. Regardless, these will be validated with analysis of the corresponding sample with the wildtype spike-in.

| **Sample ID** | **Standard** | **Spike-In** | **tp53** |  |  | |  |
| --- | --- | --- | --- | --- | --- | --- | --- |
| 1 |  |  |  |  | **KEY** | | |
| 2 |  |  |  |  | X | Het | |
| 3 |  |  |  |  |  | WT/Hom | |
| 4 |  |  |  |  | Hom | Hom, Putative | |
| 6 |  |  |  |  |  |  | |
| 7 |  |  |  |  |  |  | |
| … |  |  |  |  |  |  | |

1. Return to the plate editor view (View/Edit Plate). Exclude the standard set of samples from analysis and include the set with the wildtype spike-in. Save the changes and exit to the normal view with the four panels displayed.

**Wildtype Spike-In (0.5 µL sample: 0.5 µL WT DNA)**

**
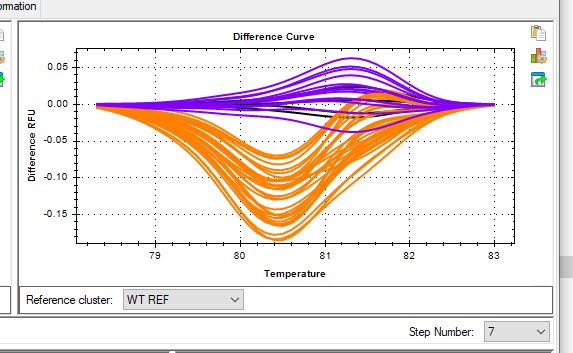
**

REF DNA

WT

Het/Hom

1. This time with the spike-in, the samples with the double deflection are either heterozygotes or homozygotes. Make sure that all samples with this deflection pattern are assigned to the same cluster. All other samples are **wildtypes.**
2. Analyze the deflection patterns of the *spike-in samples* similarly as described above in Steps 11-12, filling in the “Spike-In” column.
3. Compare the results from the analyses of the *standard* and *spike-in* runs using the following key. This parallel comparison strategy allows for *tp53* genotypes to be called with high confidence.

| **KEY** | | | |
| --- | --- | --- | --- |
| **Sample ID** | **Standard** | **Spike-In** | **tp53** |
| Fish 1 | X | X | Het |
| Fish 2 | Hom | X | Hom |
| Fish 3 |  | X | Hom |
| Fish 4 |  |  | WT |
